# Factors Associated With Health-Related Quality of Life of Parents Who Lost Their Only Child: A Cross-Sectional Study in Central China

**DOI:** 10.3389/fpubh.2020.507785

**Published:** 2020-12-02

**Authors:** Chao Rong, Li-Jie Zheng, Qian Zhang, Yi-Dan Cao, Shu-Hua Shen, Zheng-Qing Gao, Duo Wan, Wei-Wei Shen, Cai-Ming Xu, Xiao-Lan Wang

**Affiliations:** ^1^School of Humanities and Management, Zhejiang Chinese Medical University, Hangzhou, China; ^2^Bloomberg School of Public Health, Johns Hopkins University, Baltimore, MD, United States; ^3^NHC Key Laboratory of Health Economics and Policy Research (Shandong University), Jinan, China; ^4^Hangzhou West Lake District Wenxin Street Community Health Service Center, Hangzhou, China; ^5^Jiading District Center for Disease Control and Prevention, Shanghai, China; ^6^The First Affiliated Hospital of Zhejiang Chinese Medical University, Hangzhou, China; ^7^School of Economics and Management, Harbin Institute of Technology (Weihai), Weihai, China; ^8^School of Humanities and Management, Hunan Chinese Medical University, Changsha, China

**Keywords:** quality of life, EQ-5D, bereavement, EQ-VAS, who lost their only child

## Abstract

**Purpose:** The number of families who lost their only child was over one million in 2012 in China, and it is growing rapidly every year. Without their only child, they will inevitably worry about their health and life for their later years. It is important to explore the quality of life and influencing factors of the parents.

**Methods:** The cluster sampling method was adopted to select the participants in Wuhu city, Anhui province, central China. The generalized linear regression models were performed to analyze factors influencing EQ-VAS scores.

**Results:** The parents with different monthly income (*P* = 0.001) and self-rated health status (*P* < 0.001) had different EQ-VAS scores. Educational level, self-rated health status, number of chronic diseases, depression and having grandchildren were significantly associated with their EQ-VAS score.

**Conclusion:** The government should encourage public medical institutions to provide convenient health management and medical services to this vulnerable group. Priority treatment should be given to the parents who already have multiple diseases. The parents who were depressed should be given timely intervention. The government should give more financial subsidies to the parents who need to raise their grandchildren.

## Introduction

Since the late 1970s, “the one-child policy” has been the norm in the People's Republic of China, and millions of couples have been restricted in the number of children they can have. This has played a certain role in controlling the over-rapid growth of the population and promoting economic development, but it also brings about negative social phenomena. The number of families who lost their only child was over one million in 2012, and 76,000 new families were estimated to join this group each year nationwide (1). The loss of a child in a family is “the most distressing and long-lasting of all grief.” For Chinese parents, it is especially unique, because China has a traditional culture of “raising children for their old age.” Without their only child, they will inevitably worry about their health and life for their later years (2).

The pursuit of the highest standard of health is the basic right of every person. When people get old, the probability of various diseases will increase greatly. Who will take care of their health in old age after losing their only child? According to the bio-psycho-social medical model, many psychological and social factors have a great impact on health, even determinant. After the bereavement event, in addition to suffering major psychological trauma, what kind of impact will it have on the quality of life (QoL)? The introduction of the concept of QoL as an outcome measure in healthcare that emerged in the 1970s, it is the perceived quality of an individual's daily life. This includes all physical, emotional and social aspects of the individual's life (3). Therefore, it is very necessary to investigate the quality of life of the parents who lost their only child, and how best to improve their quality of life.

Yin et al. (4) pointed out that the physical status of the parents who lost their only child was much worse than that of the control group, characterized by a higher rate of chronic diseases and more hospital visits (4). Wang et al. (5) proposed that the health-related quality of life among the parents who lost their only child is lower, the length of time that had passed after losing the only-child and the personal average monthly income are the main influencing factors (5). Wang et al. (6) found that improving the senior citizens' quality of sleep and economic level, conducting health education and psychological intervention, providing facilities for sports and scientific guidance, and strengthening the treatment of chronic diseases may improve their quality of life (6). Song et al. (7) found that health-related QoL was significantly lower in bereaved family members than in controls. Bereaved family members experienced more frequent episodes of depression and suicidal thoughts. Female sex, lower income, and spousal relationship were negatively correlated with health-related QoL (8). Nicol (8) found that the time after a bereavement is related to the quality of life (7).

There were a large number of studies on the quality of life of the elderly. But there were fewer studies on the health of the parents who lost their only child. There was a study on the status of the illness. There was a study about the quality of their lives, but the EQ-VAS scale was not used. And there were some important factors that were not considered, for instance, having grandchildren, social support status, depression, and so on. This study used the EQ-VAS scale and adopted the regression analysis model to explore the quality of life and influencing factors of the parents, and to provide evidence for improving their quality of life.

## Materials and Methods

### Sampling

Data were collected from April to July 2018 in Wuhu city, Anhui province, China. The cluster sampling method was adopted to select the participants. The sampling process involved a systematic approach and a four-step scheme: (a) According to the administrative divisions of Wuhu city, it was divided into four districts: Jinghu, Yijiang, Sanshan, and Jiujiang. (b) A district was selected from the city, and it was Jinghu district. (c) All communities in the Jinghu district were selected. (d) To survey all the parents who lost their only child in these communities. The inclusion criteria were: (a) Being older than 49 (In consideration of the fact that the health bureau had brought the parents who lost their only child aged 49 or older into the systematic management, and the mothers who lost their only child aged 49 or older were no longer in the fertile period), (b) Having normal cognitive functions, and being willing and able to cooperate throughout the survey process. The exclusion criteria were: (a) The parents who lost their only child had refused to accept the government's or others' condolence or investigation, (b) Moving to another place.

Two professors from Zhejiang Chinese Medical University and 20 family planning officials in the local communities were recruited and trained as interviewers. All participants were clearly informed of the purposes of the study, and were asked to sign the consent form. All participants were assured of their rights to refuse to participate or to withdraw from the study at any time. Privacy and confidentiality of all the participants were assured. Each interview lasted for about 30 min. The criteria for determining a valid questionnaire was: The information of the three scales used were complete and accurate, and there were very few missing or no missing sociodemographic information or other data, and there were no mistakes, such as: age over 200, the participant filled in the options that were not set. Three hundred fifty participants were surveyed. There were 306 valid questionnaires, and the effective recovery rate was 87.4%.

### Measurements

#### EQ-VAS

The EuroQol visual analog scale (EQ-VAS) descriptive system was introduced by the EuroQol Group in 1990 (9)^.^ It is a 20-centimeter visual scale. Participants were asked to rate their own health for the day, with a score of 100 representing their “best health” and a score of 0 representing their “worst health” (10). It has the advantage of easy acceptance by the respondents, and is widely used in China (11–13). The EQ-VAS score of the parents who lost their only child was regarded as the dependent variable.

#### GDS

Geriatric Depression Scale(GDS) was developed by the U.S. Brink and Yesavage in 1982, which was the first depression scale designed specifically for older adults, has become widely used for depression assessment in many countries (14). It has 30 items in total. The simplified geriatric depression scale (GDS-15) is a 15-item simplified scale designed by Yesavage and Sheik in 1986, which is based on the standard version of 30 items according to the characteristics of the older adults. It was used to evaluate the depression status of the respondents in the recent week, mainly to measure the older adults feeling down, withdrawal, irritability, reduced activity, pain, and other thoughts, and negative evaluation of the past, present, and future. A higher score on the scale signifies more serious depression, and a score greater than or equal to 8 means depressive symptoms (15). A simplified scale was used in the study. GDS-15 has good reliability and discriminant validity, which can be used to measure depressive symptoms of elderly people in urban and rural areas in China (16).

#### SSRS

Shui-yuan Xiao, a Chinese scholar, developed the Social Support Rate Scale (**SSRS**), which has 10 items, and is divided into three dimensions: subjective support, objective support, and utilization of support. The total score consists of the sum of the scores of the three dimensions. A higher score means a higher level of social support. A total score of <20 signifies less social support, a total score of 20–30 signifies general social support, and a total score of more than 30 signifies satisfactory social support (17). This scale has high reliability and validity and has been widely used in China (17). The social support status was divided into two levels in this study: A score above 30 indicates good social support. Less than or equal to 30 indicates poor social support.

#### Independent Variables

The categorical independent variables included: gender (1=male, 2=female), marital status (1=living with spouses, 2=not living with spouses), having grandchildren (1=yes, 2=no). Age (1<60, 2>=60), monthly income (1 <3000, 2>=3000), social support (1=good, 2=poor), depression (1=healthy, 2=depressive). Number of chronic diseases was a continuous variable. Self-rated health status (1=good, 2=moderate, 3=bad) and educational level (1= middle school and below, 2= high school, 3= college and above) were regarded as continuous variables in the logistic regression model and generalized linear regression model.

The number of chronic diseases was surveyed by a multiple-choice question, “How many chronic diseases do you have?” Sixteen chronic diseases were listed for selection, including diabetes, hypertension, hyperlipidemia, cerebrovascular disease, a malignant tumor, coronary heart disease, Cerebral infarction (stroke), senile dementia, gynecology disease, chronic liver disease, osteoporosis, gout, asthma, arthritis, Tuberculosis (TB), hematopathy, chronic low back pain, cataract. A higher score signifies that participants have more chronic diseases.

### Quality Control

The ages of the respondents were confirmed using the household registration system. During the face-to-face investigation, the trained family planning cadres and the professors explained how to fill in the questionnaires and helped respondents complete them in their homes or community residents committee office. The database was set up by EpiData3.1 software, and double input was conducted to ensure accuracy.

### Data Analysis

Data were analyzed using the SAS version 9.1 software. Sociodemographic variables of the participants were expressed in terms of frequencies. Considering the non-normal distribution of EQ-VAS scores, the rank sum test was adopted to compare the EQ-VAS scores of the parents who lost their only child with different socio-demographic characteristics. The generalized linear regression models was performed to analyze factors influencing EQ-VAS scores.

## Results

### Characteristics of the Parents Who Lost Their Only Child

Table 1 shows characteristics of the parents who lost their only child. The median age of the parents is 62, and the median monthly income is 2,650 yuan. 46.2% of the respondents were men, and 53.8% were women. 75.7% of the respondents had an education level below middle school. 66.6% of respondents lived with their spouses. 89.3% of respondents had no grandchildren.

**Table 1:** Characteristics of the parents who lost their only child.

**Characteristics**	***M***	***Q_***R***_***
Age	62	15
Monthly income (yuan)	2,650	1,440
	*N*	%
Gender		
Male	138	46.2
Female	161	53.8
Educational level		
Middle school and below	227	75.7
High school	61	20.3
College and above	12	4.0
Marital status		
Living with spouses	195	66.6
Not living with spouses	98	33.4
Having grandchildren		
No	259	89.3
Yes	31	10.7

*7 were missing gender, 11 were missing age, 6 were missing educational level, 13 were missing marital status, 11 were missing monthly income, 16 were missing having grandchildren*.

### Comparison of EQ-VAS Scores Between Groups

The EQ-VAS score of the parent was 20~100, the median was 70, the interquartile range was 20, the average was 71, and the standard deviation was 15. Table 2 shows EQ-VAS scores comparison of the parents who lost their only child with different basic characteristics. Rank sum test results were shown: The parents with different monthly income (*P* = 0.001) and self-rated health status (*P* < 0.001) had different EQ-VAS scores. Those with a monthly income of 3,000 yuan or more had higher EQ-VAS scores than those with a monthly income of <3,000 yuan. The parents who assessed themselves as in good health and moderate health had higher EQ-VAS scores than those who assessed themselves as in poor health.

**Table 2:** The EQ-VAS score comparison of parents who lost their only child with different sociodemographic characteristics (*M* (*Q*_*R*_), score).

**Characteristic**	***N***	***M* (*Q_***R***_*)**	***Z***	***P***
Age			−1.314	0.189
<60	118	70 (30)		
≥60	177	70 (20)		
Gender			−1.016	0.310
Male	138	70 (20)		
Female	161	70 (23)		
Educational level			2.241	0.326
Middle school and below	227	70 (20)		
High school	61	75 (20)		
College and above	12	80 (20)		
Marital status			−0.648	0.517
Living with spouses	195	70 (20)		
Not living with spouses	98	80 (20)		
Monthly income (yuan)			−3.261	0.001
<3,000	195	70 (20)		
≥3,000	100	80 (10)		
Having grandchildren			−0.207	0.836
No	259	75 (14)		
Yes	31	70 (19)		
Self-rated health status			72.256	<0.001
Good	99	80 (13)		
Moderate	119	70 (10)		
Poor	38	50 (20)		

*7 were missing gender, 11 were missing age, 6 were missing educational level, 13 were missing marital status, 11 were missing monthly income, 16 were missing having grandchildren, 50 were missing self-rated health status*.

### Results of Generalized Linear Regression Analysis of EQ-VAS Score

Table 3 shows the results of generalized linear regression analysis of the factors affecting the EQ-VAS score of the parents who lost their only child. Educational level (*P* = 0.001), self-rated health status (*P* < 0.001), number of chronic diseases (*P* < 0.001), depression (*P* = 0.001), and having grandchildren (*P* = 0.001) were significantly associated with the EQ-VAS score of the parents who lost their only child.

**Table 3:** Generalized linear regression analysis of the factors affecting EQ-VAS score of the parents who lost their only child.

**Independent variables**	**Reference**	**β**	${\text{S}}_{\bar{x}}$	***P***	**OR (95%*CI*)**
Gender					
Male	Female	−0.32	0.80	0.690	0.73 (0.15 ~ 3.50)
Age					
<60	≥60	1.33	0.85	0.116	3.77 (0.72 ~ 19.77)
Educational level		2.18	0.64	0.001	8.83 (2.53 ~ 30.85)
Marital status					
Living with spouses	Not living with spouses	−0.67	0.93	0.471	0.51(0.08 ~ 3.17)
Monthly income					
<3,000	≥3,000	−0.54	0.90	0.554	0.59 (0.10 ~ 3.45)
Self-rated health status		−9.40	0.68	<0.001	0.00 (0.00 ~ 0.00)
Number of chronic diseases		−1.86	0.41	<0.001	0.16 (0.07 ~ 0.35)
Social support					
Good	Poor	0.16	0.77	0.834	1.18(0.26 ~ 5.31)
Depression					
Healthy	Depressive	3.13	0.92	0.001	22.81(3.73 ~ 139.44)
Having grandchildren					
Yes	No	2.77	0.82	0.001	15.92(3.22 ~ 78.66)

## Discussion

This study used the EQ-VAS scale and adopted the generalized linear regression models to explore the quality of life and influencing factors of the parents who lost their only child, coming from Wuhu city, Anhui province, located in central China. This study found that the parents with different monthly incomes and self-rated health statuses had different EQ-VAS scores. Educational level, self-rated health status, number of chronic diseases, depression and having grandchildren were significantly associated with their EQ-VAS score.

Huang-Hui Chen used the EQ-VAS scale to study the quality of life of the ordinary elderly in Nanjing, China. The EQ-VAS score of the elderly in Nanjing was 77.22 ± 11.12 (18). The EQ-VAS score of the parents who lost their only child was 71 ± 15. The quality of life of the parents who lost their only child was lower than that of the ordinary elderly (19). Yan Li found that these parents had difficulties in health and the government should provide support (20). Qian-Lan Yin also found these parents had a higher rate of chronic diseases than parents who have a living child (4). The bereaved family members have lower health-related QoL and mental health than the general population (7, 21, 22). Therefore, the government should encourage public medical institutions to provide convenient health management and medical services to the vulnerable group.

This study found that as self-rated health deteriorated, EQ-VAS score decreased. This reflected a high consistency between self-rated health status and quality of life. This study found that as the number of diseases increased, the EQ-VAS score decreased. This suggests that an increase in the number of diseases leads to a decrease in the quality of life. Therefore, for the parents who already have multiple diseases, priority treatment should be given.

This study found that depression was significantly associated with the EQ-VAS score. As depression levels increased, quality of life declined. This study was consistent with Yan Li's findings. Yan Li found that the parents suffered from emotional or physical health problems, and their risks of heart disease and hypertension were high, due to suffering from depression for a long time (23, 24). This is because the quality of life evaluation includes the overall evaluation of the three dimensions of body, psychology, and society. Aggravation of depression can lead to a decrease in quality of life. In addition, physical health and mental health interact with each other. Poor mental health can cause physical health deterioration. Influencing the overall quality of life. Therefore, the parents who were depressed should be given timely intervention.

This study found that the parents with grandchildren had a higher EQ-VAS score. This may be because, in Chinese culture, offspring are parents' hope and future, as well as the driving force of life. Parents with grandchildren have better mental health, which in turn improves physical health. This study found that the more educated the parents are, the higher their EQ-VAS score. Therefore, the government should give more financial subsidies to the parents who need to raise their grandchildren.

This study had several strengths. First, this was the first quantitative research we are aware of about the quality of life among parents who lost their only child in a central China city. Second, the generalized linear regression analysis models were used to explore the influencing factors of quality of life among the parents. Third, it adds to a limited number of studies on quality of life and health among parents who lost their only child, who were special vulnerable groups. This study also had the following limitations which should be acknowledged. First, this study did not analyze the data of the control group, so it is difficult to determine whether the same associated factors exist in the control group. Second, the parents were psychologically devastated by the events, and many were reluctant to be disturbed or investigated. This study only surveyed a city in Wuhu. The sample size we investigated was small, and it was difficult to represent the whole central China situation. Third, there were some possible factors that had not been investigated, such as: personality traits, the time of the event.

## Conclusions

The study quantitatively analyzed the health-related quality of life and its influencing factors among parents who lost their only child in central China city and provided some suggestions for improving the health related quality of life. Second, it can cause the government and society to pay attention to and care about the health-related quality of life of vulnerable parents. There are some topics worth further study in the future. First, we could investigate non-bereaved parents and compare the differences between the two groups. Second, we need to design specific solutions to improve the health related quality of life of parents who lost their only child.

## Data Availability Statement

The datasets generated for this study are available on request to the corresponding author.

## Ethics Statement

The studies involving human participants were reviewed and approved by The Medical Ethics Committee of Zhejiang Chinese Medical University. The patients/participants provided their written informed consent to participate in this study.

## Author Contributions

Z-QG and S-HS designed the present study. X-LW, DW, and C-MX assisted in the acquisition of subjects and data. L-JZ, Y-DC, and W-WS conducted the analysis and interpretation of data. CR and QZ prepared the manuscript. All authors contributed to and have approved the final manuscript.

## Conflict of Interest

The authors declare that the research was conducted in the absence of any commercial or financial relationships that could be construed as a potential conflict of interest.

## Abbreviations

EQ-VAS: the European quality of life visual analog scale
GDS: Geriatric Depression Scale
SSRS: Social Support Rate Scale
TB: Tuberculosis.
